# Supplementary material for: Saturation-scale functional evidence supports clinical variant interpretation in Lynch syndrome
Source: Genome Biol. 2022 Dec 22;23:266. doi: 10.1186/s13059-022-02839-z (PMC9773515; doi:10.1186/s13059-022-02839-z)
Supplement: Supplementary file 2 — Additional file 2: Figure S1. Associations with other cancers, by variant type. [file 13059_2022_2839_MOESM2_ESM.pdf]

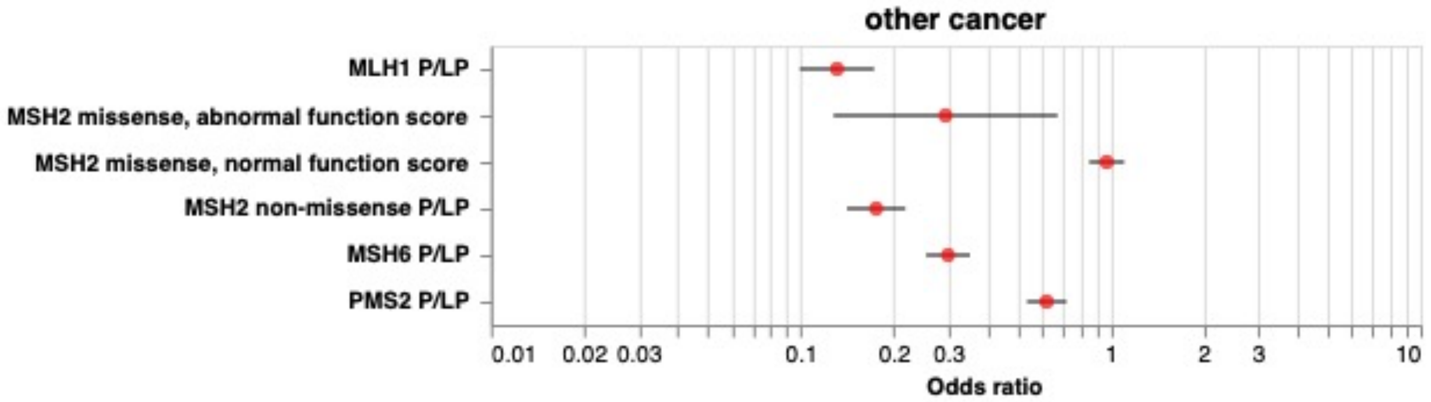

Figure S1. Associations with other cancers, by variant type. Shown as associations (odds ratio from logistic regression) between different categories of LS gene variants and cancer diagnosis, specifically excluding colorectal, uterine, and endometrial cancers.
