# Supplementary material for: Saturation-scale functional evidence supports clinical variant interpretation in Lynch syndrome
Source: Genome Biol. 2022 Dec 22;23:266. doi: 10.1186/s13059-022-02839-z (PMC9773515; doi:10.1186/s13059-022-02839-z)
Supplement: Supplementary file 5 — Additional file 5. Review history. [file 13059_2022_2839_MOESM5_ESM.docx]

Review History

**First round of review**

**Reviewer 1**

**Were you able to assess all statistics in the manuscript, including the appropriateness of statistical tests used?** Yes, and I have assessed the statistics in my report.

**Comments to author:**

The authors build upon the work in their previous deep mutational scan of MSH2 to demonstrate the utility of their data (and MAVE data in general) for patient variant reclassification. Lynch Syndrome is an interesting application area because so much is known about the disease mechanisms and associated genes. The authors use this to explore the use of MAVEs for somatic missense variant evaluation, and they describe an approach that is likely to be followed for other genes in the future.

The paper is clearly written and is a compelling success story for clinically oriented MAVEs suitable for a MAVE-focused special collection. As someone who does not have a background in clinical or medical genetics, I still found the paper compelling and easy to understand, although I have a few minor suggestions.

Major suggestions:

None

Minor suggestions:

The authors reclassify a very large number of VUS to LB, but the text focuses almost entirely on P/LP variants. I think this is worth mentioning, especially since the somatic part of the results shows that LB variants are useful for indicating that another part of the mechanism is responsible for disease. The discussion briefly mentions the problems using the PTEN data in Fayer et al. due to the lack of benign variants, but I think this is a good opportunity to point to the utility of reporting benign variants more forcefully.

The HGVS standards seem to prefer three-letter amino acid codes but the manuscript text and tables use one-letter amino acid codes (see: https://varnomen.hgvs.org/recommendations/protein/variant/substitution/). The authors should consider changing this in the tables and should definitely change the ones in the results text. In the text, the HGVS protein strings should also be encapsulated in parentheses since the actual protein was not analyzed directly, and the gene names before the ':' should be replaced with RefSeq identifiers. This will be important if the variants are automatically scraped from the manuscript text in the future.

Specific suggestions for the text:

* The abstract mentions "control variants" but this doesn't make sense out of context. I suggest changing this to "MSH2 variants".

* The abstract includes a sentence that starts with "Consistent with their pathogenicity, functionally abnormal missense variants" but I'm not sure that the "pathogenicity" of these variants can be referenced if they have not been formally classified as pathogenic.

* In the third paragraph of the introduction, it should be clarified that the one-third of VUS is specific to Lynch Syndrome. This is a lot better than many diseases!

* In the methods at the end of the "Variant classification" paragraph, it looks like two references failed to format.

* In the methods in the "Cancer association" paragraph, "python stats models package" should be "Python statsmodels package" and the software should be cited as described: https://www.statsmodels.org/stable/index.html#citation

* In the results in the second "Joint annotation of germline and somatic variants" paragraph, the authors use the terms "MSI-high" and "MIS-low" but these are not defined and I wasn't familiar with them. This could be addressed by simplifying the language removing these terms or by defining/explaining them.

**Reviewer 2**

**Were you able to assess all statistics in the manuscript, including the appropriateness of statistical tests used?** Yes, and I have assessed the statistics in my report.

**Comments to author:**

In this study, Scott, Kitzman et al used data from a recently-published deep mutational scan in the MSH2 gene (Jia et al ASHG 2021) to classify variants observed in clinical, phenotyped cancer datasets with >15k patients. Their classification method is very simple, using a threshold on the score from the prior paper, and additionally using SpliceAI predictor to address the minor subset of splice-altering variants. This classifier was quite accurate in distinguishing the (relatively small numbers) of known pathogenic and benign MSH2 variants, and thus could be used to confidently assign a pathogenicity label to ~3/4 of the considered variants of unknown significance (most benign). A somatic second-hit analysis neatly supports the use of this classification threshold. A cancer risk analysis suggests somewhat lower risk compared to truncating variants.

Overall, I think this compact, simple study is solid and well presented, and presents a clinically impactful result in terms of providing a reasonably confident classification label for many MSH2 VUSes. Other than a list of interpreted MSH2 variants, the broader scientific impact might be a bit limited however, or at least is not made clear how the methods/principles here might extend to other cancer genes or other diseases, or to provide new knowledge about mechanisms.

I cannot find any flaws and so I do not have suggestions to improve. Additional cohorts would always reasure of the results, but the available data as it is, and it does make sense to publish now, for possible impact of results, rather than waiting for more. A somatic mutation analysis might add a little bit of confidence over just the MSI label, but this would be only a marginal improvement for the study, probably.

**Authors Response**

**Point-by-point responses to the reviewers’ comments:**

**Reviewer 1:** The authors build upon the work in their previous deep mutational scan of MSH2 to demonstrate the utility of their data (and MAVE data in general) for patient variant reclassification. Lynch Syndrome is an interesting application area because so much is known about the disease mechanisms and associated genes. The authors use this to explore the use of MAVEs for somatic missense variant evaluation, and they describe an approach that is likely to be followed for other genes in the future.

The paper is clearly written and is a compelling success story for clinically oriented MAVEs suitable for a MAVE-focused special collection. As someone who does not have a background in clinical or medical genetics, I still found the paper compelling and easy to understand, although I have a few minor suggestions.

*Response: We thank the reviewer for the thoughtful critique and have addressed each of the minor suggestions below.*

Major suggestions:

None

Minor suggestions:

The authors reclassify a very large number of VUS to LB, but the text focuses almost entirely on P/LP variants. I think this is worth mentioning, especially since the somatic part of the results shows that LB variants are useful for indicating that another part of the mechanism is responsible for disease. The discussion briefly mentions the problems using the PTEN data in Fayer et al. due to the lack of benign variants, but I think this is a good opportunity to point to the utility of reporting benign variants more forcefully.

*Response: This is an excellent point - we’ve added a paragraph to the Discussion (starting with “ The large majority of MSH2 missense VUS considered here…”) to highlight that in the context of a clear phenotype, LB variants are least partially informative as they suggest the ‘causal’ functional variant is at a different locus or acts by different molecular mechanism.*

The HGVS standards seem to prefer three-letter amino acid codes but the manuscript text and tables use one-letter amino acid codes (see: https://varnomen.hgvs.org/recommendations/protein/variant/substitution/). The authors should consider changing this in the tables and should definitely change the ones in the results text. In the text, the HGVS protein strings should also be encapsulated in parentheses since the actual protein was not analyzed directly, and the gene names before the ':' should be replaced with RefSeq identifiers. This will be important if the variants are automatically scraped from the manuscript text in the future.

*Response: Thanks - updated variants to proper HGVS nomenclature*

Specific suggestions for the text:

* The abstract mentions "control variants" but this doesn't make sense out of context. I suggest changing this to "MSH2 variants".

*Response: We agree and have revised the abstract to remove the term ‘control variants’ and have added appropriate context*

* The abstract includes a sentence that starts with "Consistent with their pathogenicity, functionally abnormal missense variants" but I'm not sure that the "pathogenicity" of these variants can be referenced if they have not been formally classified as pathogenic.

*Response: Correct, not all abnormal scoring missense VUS have undergone reclassification, and we have revised this part of the abstract to avoid implying this.*

* In the third paragraph of the introduction, it should be clarified that the one-third of VUS is specific to Lynch Syndrome. This is a lot better than many diseases!

*Response: Indeed – we have qualified that sentence to reflect that it is specific to LS.*

* In the methods at the end of the "Variant classification" paragraph, it looks like two references failed to format.

*Response: Thanks-these are corrected*

* In the methods in the "Cancer association" paragraph, "python stats models package" should be "Python statsmodels package" and the software should be cited as described: https://www.statsmodels.org/stable/index.html#citation

*Response: Added*

* In the results in the second "Joint annotation of germline and somatic variants" paragraph, the authors use the terms "MSI-high" and "MIS-low" but these are not defined and I wasn't familiar with them. This could be addressed by simplifying the language removing these terms or by defining/explaining them.

*Response: We’ve retained these terms but have added a brief explanation and reference.*

**Reviewer 2**

In this study, Scott, Kitzman et al used data from a recently-published deep mutational scan in the MSH2 gene (Jia et al ASHG 2021) to classify variants observed in clinical, phenotyped cancer datasets with >15k patients. Their classification method is very simple, using a threshold on the score from the prior paper, and additionally using SpliceAI predictor to address the minor subset of splice-altering variants. This classifier was quite accurate in distinguishing the (relatively small numbers) of known pathogenic and benign MSH2 variants, and thus could be used to confidently assign a pathogenicity label to ~3/4 of the considered variants of unknown significance (most benign). A somatic second-hit analysis neatly supports the use of this classification threshold. A cancer risk analysis suggests somewhat lower risk compared to truncating variants.

Overall, I think this compact, simple study is solid and well presented, and presents a clinically impactful result in terms of providing a reasonably confident classification label for many MSH2 VUSes. Other than a list of interpreted MSH2 variants, the broader scientific impact might be a bit limited however, or at least is not made clear how the methods/principles here might extend to other cancer genes or other diseases, or to provide new knowledge about mechanisms.

I cannot find any flaws and so I do not have suggestions to improve. Additional cohorts would always reasure of the results, but the available data as it is, and it does make sense to publish now, for possible impact of results, rather than waiting for more. A somatic mutation analysis might add a little bit of confidence over just the MSI label, but this would be only a marginal improvement for the study, probably.

*Response: We thank the reviewer for the thoughtful feedback. In particular, regarding the suggestion to correlate with somatic mutation burden, we agree this would be interesting and a great idea to follow up on in the future; unfortunately, it was not assayed in this cohort.*
